# Exploring reactivity effects of self-monitoring prolonged grief reactions in daily life: A randomized waitlist-controlled trial using experience sampling methodology

**DOI:** 10.1016/j.invent.2025.100877

**Published:** 2025-10-02

**Authors:** Minita Franzen, Lonneke I.M. Lenferink

**Affiliations:** a Department of Psychology, Education & Child Studies, Erasmus University Rotterdam, P.O. Box 1738, 3000 DR, Rotterdam, the Netherlands; b Department of Psychology, Health & Technology, Faculty of Behavioural Management and Social Sciences, University of Twente, P.O. Box 217, 7500 AE, Enschede, the Netherlands; c Department of Clinical Psychology, Faculty of Social and Behavioural Sciences, Utrecht University, P.O. Box 80140, 3508 TC, Utrecht, the Netherlands; d Department of Clinical Psychology and Experimental Psychopathology, Faculty of Behavioral and Social Sciences, University of Groningen, Grote Kruisstraat 2/1, 9712 TS, Groningen, the Netherlands

**Keywords:** Prolonged grief, Bereavement, Experience sampling, Ecological momentary assessment, Loss, Reactivity

## Abstract

**Background:**

While people's symptomatology levels may change through self-monitoring of symptoms in daily life using experience sampling methodology (ESM), no controlled studies have examined such reactivity effects in the grief field.

**Objective:**

We investigated reactivity effects of self-monitoring prolonged grief reactions to determine whether self-monitoring leads to clinically significant changes in early prolonged grief disorder (PGD), post-traumatic stress disorder (PTSD), and depression symptoms at both group and individual levels.

**Methods:**

184 adults, bereaved 3 to 6 months earlier, were randomized to an ESM (*n* = 90) or waitlist condition (*n* = 94). Over two weeks, participants reported their prolonged grief reactions 5×/day. Early PGD, PTSD, and depression symptoms were assessed at baseline, post-ESM, and post-waiting. Reactivity effects on psychopathology symptom severity were examined between the ESM and waitlist group. Reliable change indices indicated clinically relevant changes in psychopathology severity and logistic regression models used to test if certain characteristics were related to the clinically relevant changes.

**Results:**

At the group level, no significant reactivity effect of self-monitoring on symptom severity for PGD, PTSD, and depression was found. Individual-level analyses indicated that most participants did not experience clinically relevant changes from pre- to post-ESM. However, people with higher baseline-PGD-scores were more likely to experience clinically relevant improvements.

**Conclusion:**

Self-monitoring prolonged grief in daily life does not seem to induce reactivity effects in symptom severity, supporting ESM as a suitable method for monitoring early prolonged grief in everyday life. Self-monitoring may benefit those with more severe initial symptoms, offering potential for targeted self-management strategies in bereavement care.

## 1 Introduction

Losing a loved one marks a universal experience. The majority of bereaved people integrate the loss into their lives without requiring professional support (Pociunaite et al., 2023; Nielsen et al., 2019). However, research indicates that approximately 10 % of those bereaved due to natural causes (Rosner et al., 2021; Lundorff et al., 2017), and notably 50 % in cases of traumatic, unnatural deaths (Djelantik et al., 2020), develop persistent and severe grief symptoms, leading to considerable distress and impairment in daily functioning. Termed Prolonged Grief Disorder (PGD; American Psychiatric Association, 2022), these enduring and debilitating manifestations of grief pose significant challenges to affected bereaved people. PGD may co-occur with symptoms of posttraumatic stress disorder (PTSD) and depression (Komischke-Konnerup et al., 2021; Heeke et al., 2023).

There is no universal response to loss. Instead, there are variations in how people experience grief, both between and within persons. Besides general between-person differences in grief reactions (e.g., one person adapts quickly while another may struggle with intense prolonged grief symptoms; Pociunaite et al., 2023; Nielsen et al., 2019), theoretical work and anecdotal accounts of bereaved people suggest that grief comes and goes in waves (e.g., Stroebe and Schut, 1999; Arizmendi and O'Connor, 2015). This points toward within-person differences in grief; grief might be a dynamic, fluctuating process and thus time- and context-dependent. Recognizing both between-person as well as within-person differences underscores the complexity and dynamic nature of grief reactions following the loss of a loved one.

To capture such dynamic and time- and context-dependent grief experiences, Experience Sampling Methodology (ESM), also referred to as Ecological Momentary Assessments (EMA, Shiffman et al., 2008; Stone and Shiffman, 1994; also see Myin-Germeys and Kuppens, 2022) can be applied. ESM involves tracking experiences, usually through self-reports, in the real world and in real-time. Typically facilitated through smartphone applications, people report on their momentary experiences, multiple times per day over an extended period of time. This way, ESM goes beyond daily-diary studies which measure experiences once a day and retrospective questionnaire-based studies that assess aggregated experiences over, for example, the past 4 weeks. Because it captures momentary experiences repeatedly in daily life, ESM allows for gaining a more ecologically valid representation of everyday grief experiences.

In essence, ESM functions as a self-monitoring tool, enabling persons to track various aspects of their daily lives. Importantly, unlike Ecological Momentary Interventions (EMIs) (cf. Teixeira, 2022), ESM is not explicitly designed to induce change in the observed experiences but merely to monitor and assess them (Eisele et al., 2022). This means that also in the context of prolonged grief, it would not be expected that self-monitoring grief reactions using ESM would lead to significant changes in grief levels or related mental health problems. Nonetheless, it has been suggested that, although not intended, the implementation of ESM may inadvertently trigger a reactivity effect (cf. Eisele et al., 2023), a systematic change of a person's behaviors and mental state (incl. emotions and symptom changes) or responses to the ESM surveys as a consequence of heightened self-awareness or attention prompted by the monitoring process (Barta et al., 2014; French and Sutton, 2010).

Reactivity effects associated with ESM can take various forms, ranging from short-term fluctuations to more stable positive or negative alterations over time. Some studies suggest that reactivity may be short-lived, with an initial spike in responsiveness during the early days of ESM implementation that gradually diminishes as people integrate the monitoring into their daily routines (e.g., Barta et al., 2014; Stone et al., 2003; Vachon et al., 2016). However, evidence also indicates more enduring positive reactivity effects following an ESM period. For instance, people may experience alleviation of depression symptoms (as evidenced in samples of depressed out-patients, Kramer et al., 2014; university students, De Vuyst et al., 2019; and rheumatology patients, Broderick and Vikingstad, 2008) and heightened feelings of empowerment (as observed in a sample of depressed out-patients; Simons et al., 2015). This positive response could stem from sustained self-awareness and self-insight, as participants engage in repeated introspection regarding their thoughts, feelings, and behaviors (Johnson et al., 2009). In the context of grief, this would be in line with the notion of self-reflective practices such as increased metacognitive awareness and expressive writing alleviating prolonged grief reactions and related symptoms (e.g., Lichtenthal and Cruess, 2010; Wagner and Maercker, 2007; Wenn et al., 2018; Wenn et al., 2019). Notably, however, positive changes due to ESM may only be temporary and may be found immediately after the ESM period but subsequently return to initial levels (cf. De Vuyst et al., 2019; Kramer et al., 2014).

Moreover, apart from questions about the stability of the observed positive reactivity, there are also questions about whether the aforementioned findings can really be attributed to the self-monitoring using ESM, as they did not compare their findings to a waitlist-control condition. For example, studies compared different ESM-protocols with each other (De Vuyst et al., 2019) or compared an ESM component added to ongoing psychological or psychopharmacological treatment with treatment as usual; Kramer et al., 2014; Simons et al., 2015). It is therefore unclear whether the observed positive reactivity effects are attributable to ESM, or to factors outside of ESM, such as the passage of time (Broderick and Vikingstad, 2008), or to other aspects of the provided treatment protocol (for more details, see the discussion sections of Kramer et al., 2014 and Simons et al., 2015).

In contrast, in the ESM field claims have also been made about a negative reactivity effect of ESM (i.e., increases in symptomatology). It has been argued, without empirically testing, that participating in ESM research, specifically when focused on negative experiences, might be perceived as too confronting and stressful (e.g., Verhagen et al., 2016; Bos et al., 2019), comparable to an initial increase in symptoms at the beginning of exposure-based trauma therapy (cf. Burger et al., 2023). Reactivity in the form of elevated distress might also explain why some ESM studies report low compliance rates (Kleiman et al., 2017). In sum, self-monitoring of daily-life experiences using ESM is not intended to change the observed experiences. Yet, findings regarding reactivity effects across ESM studies are mixed, with some suggesting positive reactivity effects rather than negative reactivity (e.g., Kramer et al., 2014; De Vuyst et al., 2019; Broderick and Vikingstad, 2008; & Simons et al., 2015; but see Conner and Reid, 2012). Importantly however, the lack of robust research designs, such as the inclusion of a waitlist-control group, prohibits us to draw firm conclusions about possible reactivity effects of ESM.

In the field of grief research, the application of ESM and the specific examination of measurement reactivity remain largely unexplored. To date, only two studies have utilized ESM to evaluate prolonged grief reactions in people's daily lives. Following a two-week ESM period assessing prolonged grief reactions five times per day in a bereaved sample with mainly non-clinical prolonged grief levels, Lenferink et al., 2022a reported that monitoring prolonged grief reactions coincided with a significant reduction in overall interview-based prolonged grief severity from before to after the ESM-phase. Similarly, Mintz et al. (2023) found small, but significant, reductions from before to after the ESM-phase for some prolonged grief reactions (e.g., psychological pain, thoughts of the deceased or about the death) when assessing these symptoms six times per day for 17 days in people with and without clinically relevant grief levels. No significant changes were found for other prolonged grief reactions (e.g., yearning, positive emotions, or thoughts about the future) both for bereaved participants with and without clinical grief levels. This suggests that monitoring one's grief reactions may lead to a positive reactivity effect and thus be helpful in reducing prolonged grief symptomatology. Importantly however, again these studies lacked a waitlist-control group (Lenferink et al., 2022a; Mintz et al., 2023) thus we cannot rule out that the reduction of prolonged grief severity was due to other reasons than using ESM.

Consequently, there is little research on reactivity effects of ESM in general, and more specifically within bereaved populations, leaving uncertainty regarding the nature and extent of reactivity in this context. To address these gaps, in the present study, we conducted a randomized controlled trial (RCT) to discern whether there is evidence for a (positive) reactivity effect of self-monitoring prolonged grief reactions. If evidence is found for a positive reactivity effect, it could imply that self-monitoring of prolonged grief reactions holds promise as an accessible tool for people struggling to cope with loss. Such a self-monitoring tool may be especially beneficial for individuals early in their grief process. Research consistently demonstrates that prolonged grief, PTSD, and depression symptoms experienced within the first six months after a loss are robust predictors of prolonged grief, PTSD, and depression levels in the future (e.g., Boelen and Lenferink, 2020; Bonanno and Malgaroli, 2020; Nielsen et al., 2019). This is why in this study, we focused on people whose loved one died 3–6 months earlier. By focusing on this critical period, self-monitoring through ESM could serve as an accessible and potentially impactful first-step intervention in mitigating the risk of prolonged and debilitating grief. Therefore, evaluating the effects of ESM in recently bereaved people holds particular significance.

In this RCT, we explored reactivity effects of self-monitoring prolonged grief reactions using ESM in recently bereaved people. Specifically, we compared the impact of self-monitoring prolonged grief reactions five times per day for two weeks with a waitlist-control condition. Importantly, in addition to assessing group-level changes in psychopathology severity (i.e., early PGD, PTSD, and depression symptoms) from pre- to post-ESM, we conducted individual-level analyses to determine possible person-specific clinically relevant changes in psychopathology levels from pre- to post-ESM. This approach allowed us to discern who experienced clinically relevant improvements (positive reactivity effect), deteriorations (negative reactivity effect), or no change in symptom severity levels. Furthermore, we explored whether background and loss-related characteristics, number of completed ESM measurements, history of psychological support, baseline psychopathology severity, and self-insight correlated with these clinically relevant changes. This enabled us to pinpoint which recently bereaved people may benefit the most from utilizing ESM-self-monitoring as an early-intervention tool. Lastly, we investigated participants' perceptions regarding the acceptability of monitoring daily prolonged grief experiences (mirroring Lenferink et al., 2022a).

Due to the scarcity of prior research in the field, our study was exploratory in nature with preregistered research aims but without a-priori hypotheses. Firstly, we investigated the extent to which psychopathology severity (i.e. early PGD, PTSD, and depression) differed (i.e., increase, decrease, or no change) after self-monitoring prolonged grief reactions using ESM compared to waitlist controls, while accounting for baseline psychopathology levels. Secondly, our aim was to calculate and compare rates of people reporting clinically relevant improvement or deterioration or no change in early PGD, PTSD, and depression severity between the ESM condition and waitlist controls. Our third aim was to identify the extent to which background and loss-related factors, number of completed ESM measurements, history of psychological support, baseline psychopathology severity, and self-insight, were related to those clinically relevant changes in psychopathology severity. Finally, given the limited research on ESM in bereaved people, we sought to amplify participants' voices by evaluating their experiences during the ESM period, particularly regarding the acceptability of participating in ESM research from the perspective of bereaved people.

## 2 Methods

### 2.1 Open science practices

This study was preregistered on the Open Science Framework (OSF) (osf.io/bvnmh) after data collection had started but before data collection was completed. At preregistration, we specified all primary and secondary outcomes as well as the analytic plan, including the use of listwise deletion for missing data (completers sample). Additional analyses on the intention-to-treat sample based on multiple imputations were not preregistered (see further details in the Statistical analyses section). An overview of all assessed measures is available online (osf.io/bvnmh). Information on the associated larger project named ‘Grief in Daily life (Grief-ID), which aims to assess and treat prolonged grief in daily life as well as data and codes used for the main analyses are available using this link: osf.io/kvfzm.

### 2.2 Design

An RCT was conducted consisting of two arms, ESM and waitlist. Because the ESM condition consisted solely of self-monitoring without active intervention components, our primary aim was to examine whether such monitoring elicited reactivity effects. A waitlist control condition was therefore selected as an ethically acceptable inactive comparison (cf. Goldberg et al., 2023). Eligible participants were randomized to either the ESM condition or waitlist control condition using a random-number service (www.random.org). The full allocation sequence was generated in advance by the last author using block randomization and a respective full randomization table created in Excel, with a 1:1 allocation ratio. Participants were assigned sequentially as they enrolled, preventing any foreknowledge of the next assignment and ensuring allocation concealment. Trained clinical psychology graduate students conducted structured telephone interviews at two or three time points depending on the condition. For the ESM condition, a pre-intervention (T1) and post-intervention (T2) interview took place. For the waitlist-control condition, a pre-waiting period (T1), post-waiting period/pre-intervention (T1b), and post-intervention (T2) interview took place. The ethics board of the Behavioral, Management, and Social Sciences (BMS) Faculty of the University of Twente approved the study (ID: 221328).

### 2.3 Participants

Dutch adults whose spouse, family member or friend died 3–6 months prior to data collection were eligible to participate. We did not include people based on prolonged grief intensity in order to obtain a heterogeneous sample. An additional inclusion criterion was owning a smartphone. People who reported high suicidal ideation (assessed at T1) were not eligible to participate. To assess suicidal ideation, we used one item from the Patient Health Questionnaire (PHQ-9; Kroenke et al., 2001) (i.e., “Over the past two weeks, how often have you been bothered by thoughts that you would be better off dead, or thoughts of hurting yourself in some way?“). If a participant answered with a score of at least 1 (“several days”), a suicide protocol was initiated. The suicide protocol consisted of the question “In the past four weeks, did you consider to end your life?”. If yes, then a follow-up question was asked “In the past four weeks, did you make a plan to end your life?” If yes, the participant was excluded from further participation and referred to their general practitioner and contact details were given for suicide-prevention support hotlines and websites. People who had ever been diagnosed with a psychotic disorder by a psychologist, therapist, or psychiatrist (assessed with single item at T1) were also not eligible to participate.

Potential participants were recruited by contacting people who completed a self-monitoring tool for PGD, i.e., the Traumatic Grief Inventory-Self Report + (TGI-SR+; Lenferink et al., 2022b) on the website www.rouwbehandeling.nl (in English ‘grief treatment’), which provides information about grief and bereavement care for the general public. This tool was introduced as a self-test to monitor whether professional bereavement care might be indicated. After completing this self-monitoring tool, the result of the test, such as whether seeking professional bereavement care might be useful, is communicated in a brief text based on the TGI-SR+ total score. People who provided consent to be contacted for future research after filling in the TGI-SR+ and who met the inclusion criteria were invited via email to participate in this RCT. Data collection took place between January 2023 and August 2023.

### 2.4 Procedure

After informed consent was obtained, the T1 interview was scheduled. Participants who met the eligibility criteria were randomly assigned to either the ESM condition or the waitlist control condition. Group allocation was communicated to them at the end of the T1 interview. Those assigned to the ESM condition received an email with instructions on how to install the Ethica (now called ‘Avicenna’) smartphone application (https://avicennaresearch.com), and the two-week ESM period began the day after the installation. Within one week following the ESM period, the post-treatment interview (T2) was conducted. For participants assigned to the waitlist condition, the post-waiting period (T1b) interview was conducted two weeks after the T1 interview. Subsequently, the waitlisted participants also started their ESM period and followed the same procedure as the ESM group which ended with the post-treatment interview (T2). No financial compensation was provided, and there were no costs associated with participating in the study.

### 2.5 Materials and measures

#### 2.5.1 Primary outcome

##### 2.5.1.1 Early prolonged grief disorder (PGD) severity (assessed at all three time points)

The primary outcome was early PGD severity according to the DSM-5-TR criteria (American Psychiatric Association, 2022). Early PGD symptoms were assessed through the TGI-CA (Lenferink et al., 2023), the interview version of the Traumatic Grief Inventory-Self Report (TGI-SR+) (Lenferink et al., 2022b). For our primary outcome variable, items representing DSM-5-TR PGD criteria were summed (i.e., item 1, 3, 6, 9, 10, 11, 18, 19, 21, and the highest answer option on item 2 or 8). On a scale from 1 (never) through 5 (always), participants reported to what extent they experienced each grief reaction during the past two weeks (e.g., “In the past two weeks, did you feel alone or detached from others?”). A score of 33 or higher indicates probable early PGD caseness (cf., Lenferink et al., 2022b). We adapted the time frame from the original TGI-CA “past month” to match the time period of the ESM-phase. The original one-month version of the TGI-CA has good internal consistency (McDonald's ω = 0.89; Lenferink et al., 2023). Cronbach's alpha for the present two-week version was 0.818 at T1, 0.866 at T1b, and 0.808 at T2, respectively, and McDonald's ω was 0.823 at T1, 0.873 at T1b, and 0.806 at T2, respectively. This suggests good and comparable reliability to the original version. However, the psychometric properties of this measure are not formally validated using a two-week time frame.

#### 2.5.2 Secondary outcomes

##### 2.5.2.1 PTSD severity (assessed at all three time points)

PTSD symptoms were assessed with the PTSD-Checklist for DSM-5 (PCL-5; Blevins et al., 2015; Boeschoten et al., 2014). The PCL-5 consists of 20 statements, which are rated on a 5-point Likert scale from 0 (not at all) to 4 (extremely). Similarly to the PGD measure, the time frame was adjusted from “past month” to “past two weeks” to fit the ESM period. In addition, given the administration via telephone, statements were phrased as questions, and ‘stressful event’ was changed to ‘death of loved one’ (e.g., “In the past two weeks, how much were you bothered by taking too many risks or doing things that could cause you harm?”). A total score was calculated by summing all 20 questions, with higher scores being indicative of more severe PTSD levels (range 20–100). A total score of 33 or higher was used as an indicator for probable PTSD (Weathers et al., 2013; Krüger-Gottschalk et al., 2017). The PCL-5 has shown to have good reliability and validity (Blevins et al., 2015). The PCL-5 demonstrated a good internal consistency at all three time points (T1 α = 0.871, T1b α = 0.889, and T2 α = 0.881).

##### 2.5.2.2 Depression severity (assessed at all three time points)

Depression symptoms were assessed with the Patient Health Questionnaire (PHQ-9; Kroenke et al., 2001). On a 4-point Likert scale from 0 (not at all) to 3 (nearly every day), participants indicated how often they were bothered by each of the nine symptoms during the past two weeks. The formulation of the items as presented in the original questionnaire was altered from statements into questions (e.g., “Over the past two weeks, how often have you been bothered by having little interest or pleasure in doing things?”). The PHQ-9 is a psychometrically sound measure of depression severity (Kroenke et al., 2001). Based on Kroenke et al. (2001), a total score of at least 10 was used as a cut-off for clinical depression. The PHQ-9 demonstrated a good internal consistency at all three time points (T1 α = 0.772, T1b α = 0.777, and T2 α = 0.763).

#### 2.5.3 Other measures

##### 2.5.3.1 Background characteristics (assessed at T1)

Background characteristics were assessed with questions about gender (1 (male), 2 (female), 3 (other)), date and country of birth, and highest obtained level of education (0 (primary school), 1 (high school), 2 (vocational education), 3 ((applied) university).

##### 2.5.3.2 Loss-related characteristics (assessed at T1)

Questions related to the loss of their loved one consisted of the date of death, the relationship toward the lost person (0 (partner), 1 (child), 2 (parent), 3 (sibling), 4 (grandparent), 5 (grandchild), 6 (friend), 7 (other, namely)), the cause of death (0 (physical illness), 1 (accident), 2 (suicide), 3 (homicide/manslaughter), 4 (other, namely)), to what extent this loss was unexpected (1 (completely expected) to 5 (completely unexpected)), if they ever received psychological support prior to the loss (0 (no), 1 (yes)), if they ever received professional grief support related to the loss (0 (no), 1 (yes), and if they responded yes then if they currently (i.e., at the time of the interview) received professional grief support related to the loss (0 (no), 1 (yes)). In case a participant indicated to have experienced multiple losses in the previous 3–6 months, they were asked to answer all questions about the loss they considered to be the most significant loss.

##### 2.5.3.3 Self-insight (assessed at all three time points)

The Self-Reflection and Insight Scale (SRIS) (Grant et al., 2002) was used to assess self-reflection (12 items) and insight (8 items). Example items of both subscales are “I frequently take time to reflect on my thoughts” and “I usually know why I feel the way I do”, respectively. Answers were provided on a 6-point Likert scale from 1 (strongly disagree) to 6 (strongly agree). The SRIS shows good psychometric qualities (Grant et al., 2002; Silvia, 2022). The SRIS demonstrated a good internal consistency at all three time points for both the total scale (T1 α = 0.865, T1b α = 0.921, and T2 α = 0.899), as well as the self-reflection subscale (T1 α = 0.900, T1b α = 0.937, and T2 α = 0.937) and the self-insight subscale (T1 α = 0.805, T1b α = 0.828, and T2 α = 0.800).

##### 2.5.3.4 Acceptability of participating in ESM research (assessed at T2)

To assess the acceptability of self-monitoring prolonged grief reactions in daily life, we used the subscale Personal Benefits (i.e., four items e.g., “I found participating in daily diary measures in the app beneficial to me.”) and the subscale “Emotional Reactions” (i.e., four items e.g., “Participating in daily diary measures in the app raised emotional issues for me that I had not expected.”) from the Reactions to research participation questionnaire (RRPQ; Newman et al., 2001). Following Lenferink et al., 2022a, we referred to “participating in daily diary measures in the app” instead of “study” and statements were rephrased as questions. Answers were rated a scale from 1 (strongly disagree) to 5 (strongly agree). Sum scores were created for each subscale. Psychometric properties of the original and adapted version are considered sufficient (Newman et al., 2001; Waterman et al., 2021). In the present study, Cronbach's alpha levels were 0.853 for Personal Benefits subscale and 0.824 for Emotional Reactions subscale.

#### 2.5.4 Intervention

During the ESM-phase, participants received five semi-random beeps per day on 14 consecutive days. More specifically, on each day, beeps occurred randomly between 8.30 am–9.30 am, 11.30 am–12.30 pm, 2.30 pm–3.30 pm, 5.30 pm–6.30 pm, and 8.30 pm–9.30 pm. After 10 and 20 min, a reminder was sent when people had not yet completed the ESM-survey. Participants had 60 min to complete the ESM-questionnaire. Each questionnaire consisted of a maximum of 17 items. Completion of these items took approximately 1–2 min. To increase compliance, reminder emails were sent when participants missed more than 50 % (i.e., ≥3) of the surveys on one day. These reminders were only sent once during the study duration.

##### 2.5.4.1 ESM questionnaire

Mirroring Lenferink et al., 2022a, prolonged grief reactions in daily life were assessed with 11 items that map onto the criteria of the DSM-5-TR for PGD and were developed using cognitive interviewing with ESM and/or grief experts (see Table 1). Participants rated their prolonged grief reactions in the past 3 h on a 7-point Likert scale, ranging from 0 (not at all) to 6 (very much). Additionally, the questionnaire included contextual items to assess the time of the day, physical environment (i.e., home, school, work, other), social environment (i.e., alone, with one other person, with multiple others), if not alone, type of relationship with the other person (i.e., romantic partner, child, parent, sibling, friend, acquaintance, colleague/fellow student, other), and quality of the social contact (i.e., from 0 (very unpleasant) to 6 (very pleasant)).

**Table 1:** Overview of ESM items to assess daily prolonged grief reactions.

In the past three hours…
I found myself yearning for him/her.
I had intrusive thoughts or images related to the person who died.
It felt as if a part of me has died along with the deceased.
It felt unreal that he/she is dead.
I avoided places, objects, or thoughts that reminded me that he/she is dead.
I felt sad because of his/her death.
I felt bitterness or anger because of his/her death.
It was difficult for me to do something (e.g., social activities, studying, working, sports, hobbies) because of his/her death.
I felt emotionally numb because of his/her death.
I felt that life is unfulfilling or meaningless without him/her.
I felt alone or detached from other individuals because of his/her death.

### 2.6 Statistical analyses

**Fig. 1:**
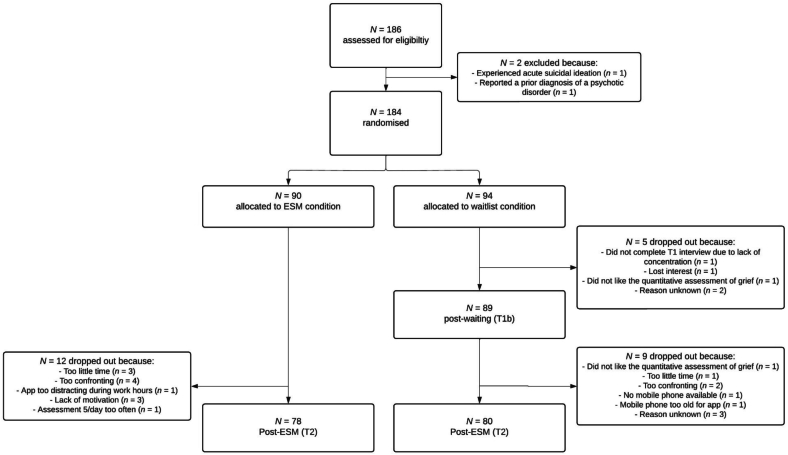
Flowchart of participants.

As stated in the pre-registration (osf.io/bvnmhn), a sample size calculation was conducted a priori, which indicated that a sample of 128 participants would be sufficient to detect a moderate effect size with a power of 80 % to identify a difference between the two conditions. To account for a dropout rate of 36 % (cf. Lenferink et al., 2022a), we aimed to recruit 200 participants. A total of 186 started our study (see Fig. 1). Analyses were conducted in Mplus version 8 (Muthén and Muthén, 1998-2017) and SPSS version 28 (IBM Corp., 2021). The significance level was set at α = 0.05.

Differences between the ESM and waitlist control condition in terms of age (in years), and early PGD, PTSD, and depression symptoms assessed at T1 were tested using independent samples *t*-tests and Mann-Whitney *U* tests. Chi-square tests were used to examine group-differences in gender (0 = male, 1 = female; one person self-identifying as non-binary not included), educational level (0 = primary, secondary or vocational, 1 = college/university), kinship to deceased (0 = spouse or child, 1 = other), and cause of death (0 = illness, 1 = other).

To answer research question 1, reactivity effects in terms of changes in psychopathology symptom severity were examined between the ESM and waitlist group using three separate analyses of covariance (ANCOVAs). The dependent variables were mean symptom levels of early PGD, PTSD, or depression at post-intervention (T2)/post-waiting period (T1b). The independent variable was condition (ESM vs. waitlist control). T1 symptom levels of early PGD, PTSD, or depression were included as covariates, respectively. These ANCOVAs were performed on the completers sample (*n* = 155) in line with the pre-registered approach of list-wise deletion for cases with missing data. We then repeated the analyses on the intention-to-treat sample (*N* = 184) using multiple imputation to handle missing data. While not pre-registered, multiple imputation implemented in Mplus was used to impute missing data by generating 100 datasets. The proportion of missing outcome data at T2 was 15.8 % (*n* = 29). Logistic regression models showed that missingness was not systematically associated with demographic, loss-related, or baseline psychopathology characteristics, supporting the assumption that data were missing at random and the appropriateness of multiple imputation (see Supplementary Table 1). Effect sizes (Cohen's *d*) for differences within and between groups were computed. The completers sample (*n* = 155) consisted of all *n* = 89 people in the waitlist control group who completed T1 and T1b and *n* = 66 people in the ESM group (out of *n* = 78) who completed T1 and T2 and fulfilled our pre-registered criterion of ≥50 % of completed ESM measurements. Based upon reviewer request regarding potential bias due to within-person missingness in the ESM assessments, we repeated our ANCOVAs including the number of completed ESM measurements as a covariate.^1^

To answer research question 2, reliable change indices (RCIs) were computed, to determine clinically relevant changes in early PGD, PTSD, and depression severity from before (pre-intervention/pre-waiting; T1) to after ESM (post-intervention; T2) or waiting (post-waiting; T1b). For calculating RCIs, the formula from Jacobson and Truax (1991) was used (in line with earlier work; e.g., Lenferink et al., 2019): $\mathrm{RCI}=\frac{X2-X1}{\surd \mathrm{Sdiff}}$. X2 represents a participant's post-intervention (T2)/post-waiting period (T1b) score and X1 represents a participant's pre-intervention/pre-waiting score (T1 for both conditions). Sdiff is computed by using Cronbach's alpha and standard deviation of the T1 scores. An RCI > 1.96 was considered a clinically relevant change, meaning the change was unlikely due to chance (*p* < .05; cf. Jacobson and Truax, 1991). Subsequently, the number of participants reporting a reliable change was counted and percentages between the two conditions were compared using Chi-square tests.

To answer research question 3, again RCIs were computed. This time for determining clinically relevant changes in early PGD, PTSD, and depression severity from pre-ESM (i.e., T1 for ESM-group and T1b for waitlist controls) to post-ESM (T2 for both groups) for all participants with complete data (*n* = 137). Subsequently, based on their RCIs, participants were assigned to one of the following groups: 1) no clinically relevant change in early PGD, PTSD, and depression symptom severity, 2) clinically relevant improvement in early PGD, PTSD, or depression symptom severity, 3) clinically relevant deterioration in early PGD, PTSD, or depression symptom severity. We subsequently used the membership of those groups to test if certain characteristics were related to the clinically relevant changes. In a multinomial logistic regression, membership of “RCI-groups” represented the dependent variable with ‘no change’ being the reference group. In separate univariate models, the following independent variables were included: background and loss-related characteristics (i.e., gender, age, education, time since loss, kinship, expectancy, and cause of death), number of completed ESM measurements, current grief support at T1, baseline psychopathology severity, and self-insight at T1. Based on relevant correlates (i.e., variables that were significantly (*p* < .05) related to the outcome in univariate analyses) it was planned to then include those correlates simultaneously in a multivariate logistic regression model. However, as only one significant correlate emerged, the multivariate model was deemed redundant. We note that, in line with our preregistration and hypothesis-generating approach, no correction for multiple testing was applied, which increases the risk of inflated Type I error. Accordingly, these findings should be interpreted with caution.

Finally, to answer research question 4, we followed the approach of Lenferink et al., 2022a to examine how participants considered the acceptability of self-monitoring prolonged grief reactions. We evaluated descriptive statistics of both RRPQ subscales and ran correlation analyses between the scores on the RRPQ subscales and early PGD symptom severity levels at pre-ESM (T1/T1b) and post-ESM (T2).

## 3 Results

### 3.1 Sample characteristics and preliminary analyses

The T1 interview was started with a total of 186 people. Two participants were excluded (and the interview not completed) because they met our exclusion criteria. Specifically, one person experienced acute suicidal ideation and one person reported a prior diagnosis of a psychotic disorder. This means that 184 participants represent the intention-to-treat sample and were randomly allocated to the ESM intervention group (*n* = 90) or the waitlist control group (*n* = 94). One participant of the waitlist group did not complete the T1 interview and subsequently decided to drop out of the study due to lack of concentration.

Participants (*N* = 184) were on average 55 years old (*SD* = 12.12; range 22–85). The majority of participants were born in the Netherlands (92 %), self-identified as woman (*n* = 152; 31 as man, 1 non-binary), and had a (applied) university degree (62 %). Most participants lost a spouse (48 %) or parent (33 %), mostly due to illness (78 %). Fifty-five participants (30 %) received concurrent professional psychological support related to their loss at T1. For an overview of socio-demographic and loss-related characteristics and symptom-levels of early PGD, PTSD, and depression for the entire sample and per condition, see Table 2.

**Table 2:** Background and loss-related characteristics and psychopathology symptom levels for the total sample and per condition at all three time points.

Characteristic	Total sample (*N* = 184)		ESM condition (*n* = 90)		Waitlist condition (*n* = 94)	
T1						
Gender, *N* (%)						
Woman	152	(82.6)	71	(78.9)	81	(86.2)
Man	31	(16.8)	18	(20.0)	13	(13.8)
Non-binary	1	(0.5)	1	(1.1)	0	(0)
Age (in years), *M* (*SD*)	54.56	(12.12)	53.89	(13.24)	55.19	(10.97)
Country of birth, *N* (%)						
The Netherlands	170	(92.4)	85	(94.4)	85	(90.4)
Germany	5	(2.7)	3	(3.3)	2	(2.1)
Belgium	4	(2.2)	1	(1.1)	3	(3.2)
Other	4	(2.7)	1	(1.1)	4	(4.3)
Education, *N* (%)						
Primary school	0	(0)	0	(0)	0	(0)
High school	15	(8.2)	6	(6.7)	9	(9.6)
Vocational education	54	(29.5)	31	(34.4)	23	(24.5)
(Applied) University	114	(62.3)	53	(58.9)	62	(66.0)
Time since loss (in weeks), *M* (*SD*)	20.28	(5.19)	20.53	(4.85)	20.04	(5.51)
Kinship (the deceased is my…) *N* (%)						
Spouse	88	(47.8)	41	(45.6)	47	(50.0)
Child	15	(8.2)	7	(7.8)	8	(8.5)
Parent	61	(33.2)	32	(35.6)	29	(30.9)
Sibling	9	(4.9)	5	(5.6)	4	(4.3)
Grandparent	1	(0.5)	1	(1.1)	0	(0)
Grandchild	1	(0.5)	0	(0)	1	(1.1)
Friend	3	(1.6)	0	(0)	3	(3.2)
Other	6	(3.3)	4	(4.4)	2	(2.1)
Expectancy of death, *M* (*SD*)	3.22	(1.58)	3.16	(1.55)	3.29	(1.62)
Cause of death, *N* (%)						
Physical illness	143	(77.7)	69	(76.7)	74	(78.7)
Accident	10	(5.4)	8	(8.9)	2	(2.1)
Suicide	10	(5.4)	6	(6.7)	4	(4.3)
Homicide	0	(0)	0	(0)	0	(0)
Other	21	(11.4)	7	(7.8)	14	(14.9)
Prior mental health support – unrelated to loss, *N* (%)						
Yes	98	(53.3)	48	(53.3)	50	(53.2)
No	86	(46.7)	42	(46.7)	44	(46.8)
Prior mental health support related to loss – prior grief support, *N* (%)						
Yes	74	(40.2)	34	(37.8)	40	(42.6)
No	110	(59.8)	56	(62.2)	54	(57.4)
Current mental health support related to loss – current grief support, *N* (%)						
Yes	55	(29.9)	26	(28.9)	29	(30.9)
No	129	(70.1)	64	(71.1)	65	(69.1)
Early PGD symptom-level, *M* (*SD*)	29.70	(7.29)	29.71	(7.29)	29.68	(7.33)
Early PGD caseness, *N* (%)						
Yes	69	(37.5)	34	(37.8)	35	(37.2)
No	115	(62.5)	56	(62.2)	59	(62.8)
Depression symptom-level, *M* (*SD*)	9.89	(5.19)	9.52	(5.34)	10.23	(5.03)
Depression caseness, *N* (%)						
Yes	97	(52.7)	45	(50.0)	52	(55.3)
No	87	(47.3)	45	(50.0)	42	(44.7)
PTSD symptom-level, *M* (*SD*)^A^	21.51	(12.12)	21.43	(12.60)	21.58	(11.71)
PTSD caseness, *N* (%)						
Yes	36	(19.7)	19	(21.1)	17	(18.3)
No	147	(80.3)	71	(78.9)	76	(81.7)
Self-insight total, *M* (*SD*)^A^	90.17	(13.72)	87.91	(14.35)	92.37	(12.77)
Self-reflection subscale, *M* (*SD*)^A^	54.99	(10.64)	52.94	(11.62)	56.97	(9.25)
Self-insight subscale, *M* (*SD*)^A^	35.18	(6.79)	34.97	(6.69)	35.40	(6.92)
T1b					*n* = 89	
Early PGD symptom-level, *M* (*SD*)	–	–	–	–	27.40	(7.68)
Early PGD caseness, *N* (%)						
Yes	–	–	–	–	24	(27.00)
No	–	–	–	–	65	(73.00)
Depression symptom-level, *M* (*SD*)	–	–	–	–	8.63	(4.68)
Depression caseness, *N* (%)						
Yes	–	–	–	–	33	(37.10)
No	–	–	–	–	56	(62.90)
PTSD symptom-level, *M* (*SD*)	–	–	–	–	19.81	(12.09)
PTSD caseness, *N* (%)						
Yes	–	–	–	–	11	(12.40)
No	–	–	–	–	78	(87.60)
Self-insight total, *M* (*SD*)	–	–	–	–	92.38	(15.26)
Self-reflection subscale, *M* (*SD*)	–	–	–	–	56.91	(11.08)
Self-insight subscale, *M* (*SD*)	–	–	–	–	35.47	(6.57)


Characteristic	Total sample *N* = 158		ESM condition *n* = 78		Waitlist condition *n* = 80	
T2						
Early PGD symptom-level, *M* (*SD*)	26.41	(6.59)	26.82	(6.19)	26.01	(6.97)
Early PGD caseness, *N* (%)						
Yes	29	(18.4)	16	(20.5)	13	(16.3)
No	137	(81.6)	62	(79.5)	67	(83.8)
Depression symptom-level, *M* (*SD*)	7.77	(4.61)	7.86	(4.90)	7.68	(4.34)
Depression caseness, *N* (%)						
Yes	51	(32.3)	25	(32.1)	26	(32.5)
No	107	(67.7)	53	(67.9)	54	(67.5)
PTSD symptom-level, *M* (*SD*)	18.65	(11.43)	19.63	(11.84)	17.7	(11.00)
PTSD caseness, *N* (%)						
Yes	21	(13.3)	13	(16.7)	8	(10.0)
No	137	(86.7)	65	(83.3)	72	(90.0)
Self-insight total, *M* (*SD*)	90.37	(14.48)	89.14	(14.42)	91.58	(14.52)
Self-reflection subscale, *M* (*SD*)	55.16	(11.60)	54.26	(12.17)	56.04	(11.03)
Self-insight subscale, *M* (*SD*)	35.22	(6.20)	34.88	(6.38)	35.54	(6.04)
Completed ESM measurements, *M* (*SD*)	47.17	(21.56)	45.39	(23.19)	48.97	(19.74)

*Note*. PGD = prolonged grief disorder; PTSD = posttraumatic stress disorder; T1 = pre-ESM or pre-waiting assessment; T1b = post-waiting assessment; T2 = post-ESM assessment. The cut-off for clinical caseness was a score of 33 or higher for early PGD, 33 or higher for PTSD, and 10 or higher for depression.

A For the respective variable, the total sample is based on *n* = 183 and the waitlist on *n* = 93 given that one person decided to terminate the T1 interview early and did not complete the entire interview.

At T1, 38 % of the total sample scored above the clinical cutoff indicating early PGD, 20 % indicated PTSD, and 53 % indicated depression. Rates were comparable between the ESM condition (38 % early PGD, 22 % PTSD, and 50 % depression caseness) and the waitlist condition (37 % early PGD, 18 % PTSD, and 55 % depression caseness).

Participants in the two conditions did not significantly differ with respect to background and loss-related characteristics; see Table 3.

**Table 3:** Testing differences in baseline (T1) characteristics and PGD, PTSD, and depression symptoms between the ESM- and waitlist-control condition using chi-square tests, independent sample *t*-tests, and Mann-Whitney U tests (*N* = 184).

Characteristic	Test	*df*	*p*
Gender	χ^2^ = 1.329	1	0.249
Education	χ^2^ = 0.980	1	0.322
Age	*t* = −0.727	182	0.468
Kinship to deceased	χ^2^ = 0.309	1	0.578
Cause of death	χ^2^ = 0.490	1	0.484
Symptom-levels (T1)			
TGI-CA (Early PGD)	*t* = 0.028	182	0.978
PCL-5 (PTSD)	*z* = −0.130		0.897
PHQ-9 (Depression)	*z* = −0.953		0.341

*Note.* TGI-CA = Traumatic Grief Inventory – Clinician Administered; PCL-5 = PTSD Checklist for DSM-5; PHQ-9 = Patient Health Questionnaire-9; PGD = prolonged grief disorder; PTSD = posttraumatic stress disorder; *df* = degrees of freedom; T1 = pre-ESM or pre-waiting assessment. Based on a significant Shapiro-Wilk test, a non-normal distribution was assumed for the PCL-5 and PHQ-9 variable and a Mann-Whitney *U* test was performed instead of an independent sample *t*-test.

Based on all participants that have started the ESM-phase (i.e., *n* = 179 for both conditions combined; *n* = 90 in the ESM condition and *n* = 89 in the waitlist condition), the mean value of completed ESM measurements was 47 (*SD* = 22; min = 0, max = 70). This represents an absolute compliance rate of 67 %. In terms of retention rate, 139 participants (78 %) completed at least 50 % of the measurements.

### 3.2 Differences in early PGD, PTSD, and depression severity between the ESM and waitlist condition (aim 1)

Based on the intention-to-treat sample (*N* = 184), the ANCOVAs indicated no significant differences in early PGD, PTSD, and depression severity after ESM (*n* = 90) or waiting period (*n* = 94) between the conditions when taking early PGD, PTSD, or depression severity at T1 into account. Table 4 shows the parameter estimates.

**Table 4:** Estimated parameters for analyses of covariance comparing the ESM and waitlist condition in the intention-to-treat sample (*N* = 184).

	Early PGD at T2/T1b		PTSD at T2/T1b		Depression at T2/T1b	
	*B*	*SE*	*B*	*SE*	*B*	*SE*
Intercept	3.41	2.47	2.15	3.17	0.58	1.61
Condition	0.82	1.15	0.17	1.70	0.47	0.77
Symptom intensity at T1	0.76^⁎⁎⁎^	0.06	0.81^⁎⁎⁎^	0.05	0.69^⁎⁎⁎^	0.05

*Note.* PGD = prolonged grief disorder; PTSD = posttraumatic stress disorder; T1 = pre-ESM or pre-waiting assessment; T1b = post-waiting assessment; T2 = post-ESM assessment; *B* = unstandardized beta; *SE* = standard error. ^⁎^*p* < .05, ^⁎⁎^*p* < .01, ^⁎⁎⁎^*p* < .001.

Based on the completers sample (*n* = 155), again the ANCOVAs did no show significant differences in early PGD, PTSD, and depression severity after ESM (*n* = 66) or waiting period (*n* = 89) between the conditions when taking early PGD, PTSD, or depression severity at T1 into account (see Supplementary Table 3).

### 3.3 Clinically relevant changes in early PGD, PTSD, and depression severity from T1 (pre-intervention/pre-waiting) to post-ESM (T2) or post-waiting (T1b) (aim 2)

#### 3.3.1 Early PGD severity

Regarding early PGD severity, for the ESM-condition, 52 out of 66 people (79 %) reported no clinically relevant change in early PGD severity from T1 to T2. Twelve people (18 %) reported a clinically relevant improvement and two persons (3 %) reported a clinically relevant worsening in early PGD severity.

For the waitlist-condition, 75 out of 89 people (84 %) reported no clinically relevant change in early PGD severity from T1 to T1b. Eleven people (13 %) reported a clinically relevant improvement and three persons (3 %) reported a clinically relevant worsening in early PGD severity.

The rate of people reporting clinically relevant improvement in early PGD severity did not significantly differ from the rate of people reporting no change or clinically relevant worsening in early PGD severity (χ^2^(1) = 1.02, *p* = .313). Too few people reported clinically relevant worsening of symptoms. We therefore merged these people with the group of people who reported no change.

#### 3.3.2 PTSD severity

Regarding PTSD severity, for the ESM-condition, 60 out of 66 people (90 %) reported no clinically relevant change in PTSD severity from T1 to T2. Three people (5 %) reported a clinically relevant improvement and three persons (5 %) reported a clinically relevant worsening in PTSD severity.

For the waitlist-condition, 81 out of 89 people (91 %) reported no clinically relevant change in PTSD severity from T1 to T1b. Five people (6 %) reported a clinically relevant improvement and three persons (3 %) reported a clinically relevant worsening in PTSD severity.

The rate of people reporting clinically relevant improvement in PTSD severity did not significantly differ from the rate of people reporting no change or clinically relevant worsening in PTSD severity (χ^2^(1) = 0.09, *p* = .765). Too few people reported clinically relevant worsening of symptoms. We therefore merged these people with the group of people who reported no change.

#### 3.3.3 Depression severity

Regarding depression severity, for the ESM-condition, 62 out of 66 people (94 %) reported no clinically relevant change in depression severity from T1 to T2. Four people (6 %) reported a clinically relevant improvement and no person reported a clinically relevant worsening in depression severity.

For the waitlist-condition, 84 out of 89 people (94 %) reported no clinically relevant change in depression severity from T1 to T1b. Five people (6 %) reported a clinically relevant improvement and no person reported a clinically relevant worsening in depression severity.

The rate of people reporting clinically relevant improvement in depression severity did not significantly differ from the rate of people reporting no change or clinically relevant worsening in depression severity (χ^2^(1) = 0.01, *p* = .907).

### 3.4 Possible correlates predicting clinically relevant changes in early PGD, PTSD, and depression severity from before (T1 or T1b) to after (T2) ESM (aim 3)

At this point, we merged the T1 data of participants from the ESM-condition with the T1b data from the waitlist-condition to examine clinically relevant changes from pre- to post-ESM for the entire sample (i.e., *n* = 137).

#### 3.4.1 Early PGD severity

Regarding early PGD severity, 111 out of 137 people (81 %) reported no clinically relevant change in early PGD severity from pre-ESM to post-ESM. Nineteen people (14 %) reported a clinically relevant improvement and seven persons (3 %) reported a clinically relevant worsening in early PGD severity.

#### 3.4.2 PTSD severity

Regarding PTSD severity, 127 out of 137 people (93 %) reported no clinically relevant change in PTSD severity from pre-ESM to post-ESM. Six people (4 %) reported a clinically relevant improvement and four persons (3 %) reported a clinically relevant worsening in PTSD severity.

#### 3.4.3 Depression severity

Regarding depression severity, 129 out of 137 people (94 %) reported no clinically relevant change in depression severity from pre-ESM to post-ESM. Seven people (5 %) reported a clinically relevant improvement and one person (1 %) reported a clinically relevant worsening in depression severity.

Next, based on those clinically relevant change results, participants were assigned to either of the following groups. Out of 137 participants, 104 (76 %) showed no clinically relevant change in neither early PGD, PTSD, nor depression symptoms (i.e. ‘no change group’), 23 (17 %) experienced improvement in at least one measure (i.e. ‘improvement group’), while 10 (7 %) showed deterioration in at least one measure (i.e. ‘deterioration group’). No participants experienced improvement in one outcome while deterioration in another.

Given the small group size of only 10 individuals in the deterioration group, we combined the deterioration group with the no change group. This combined group (*n* = 114) served as the reference group with which the improvement group was compared. Given the dichotomous nature of this variable, univariate binary logistic regression analyses were performed to determine possible correlates predicting clinically relevant change. See Table 5 for test statistics.

**Table 5:** Estimates for covariates predicting likelihood of belonging to group with clinically relevant improvement (*n* = 23) compared to no change or deterioration (combined group; *n* = 114).

Covariate	*B*	*SE*	*p*	*OR*	95 % CI	
					*LL*	*UL*
Gender	0.921	0.777	0.236	2.511	0.547	11.517
Age	0.002	0.019	0.915	1.002	0.966	1.039
Education	−0.060	0.469	0.899	0.942	0.376	2.362
Time since loss	0.020	0.046	0.658	1.020	0.933	1.116
Kinship	−0.721	0.490	0.141	0.486	0.186	1.271
Expectancy	0.206	0.155	0.182	1.229	0.908	1.664
Cause of death	−0.236	0.596	0.692	0.789	0.245	2.540
Completed ESM moments	0.002	0.028	0.941	1.002	0.949	1.058
Current grief support	0.724	0.840	0.389	2.062	0.398	10.699

Symptom-levels (T1)						
PGD	0.092	0.038	**0.017**	1.096	1.017	1.181
PTSD	0.024	0.020	0.212	1.025	0.986	1.065
Depression	0.084	0.045	0.061	1.088	0.996	1.189
Self-insight total	−0.019	0.018	0.281	0.981	0.947	1.016
Self-reflection subscale	−0.022	0.024	0.371	0.979	0.933	1.026
Self-insight subscale	−0.025	0.032	0.436	0.975	0.915	1.039

*Note*. The following variables were dichotomized: education ((applied) university vs. primary, secondary, and vocational education), kinship (spouse or child vs. other), cause of death (natural vs. unnatural). PGD = prolonged grief disorder; PTSD = posttraumatic stress disorder; T1 = pre-ESM or pre-waiting assessment; *B* = unstandardized beta; *SE* = standard error; OR = odds ratio; CI = confidence interval; LL = lower limit; UL = upper limit.

The only significant predictor for clinically significant improvement was early PGD severity at T1 (*B* = 0.092, *SE* = 0.038; *p* = .017; *OR* = 1.096). People with higher PGD baseline scores were more likely to be experiencing clinically relevant improvements compared to no change or deterioration. For a visual presentation of the predicted probability of experiencing a clinically relevant improvement (compared to no change or deterioration) by T1 PGD score see Supplementary Fig. 1. For a person with a PGD score of 33 (representing the cut-off for an indication of early PGD caseness), the likelihood of a clinically relevant improvement is 20.3 %. Sensitivity analyses run with the original grouping variable including 3-groups (i.e., no change, improvement, deterioration) resulted in comparable findings (see Supplementary Table 4 for all details). Early PGD severity at T1 was a significant predictor of clinically relevant improvement (*B* = 0.090, *SE* = 0.038; *p* = .019; *OR* = 1.094) compared to no change. More specifically, people with early PGD severity at T1 were more likely to be experiencing clinically relevant improvements compared to no change. In addition, baseline depression severity was also a significant predictor of clinically relevant improvement compared to no change (*B* = 0.095, *SE* = 0.046; *p* = .039; *OR* = 1.005). More specifically, people with higher depression baseline scores were more likely to be experiencing clinically relevant improvements compared to no change.

### 3.5 Acceptability of monitoring daily prolonged grief experiences (aim 4)

**Fig. 2:**
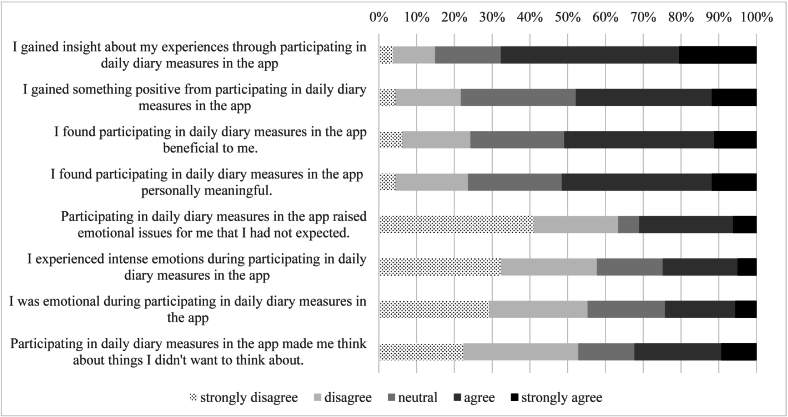
Extent of agreement with reactions to participating in the daily diary measures in the app (*n* = 161).

At T2, 161 people completed the RRPQ providing information about to what extent they have experienced personal benefits and emotional reactions due to their participation in daily diary measures in the app. As per the four items representing personal benefits, the mean item score was 3.43 (*SD* = 0.88), which reflects a neutral response (i.e., answer option 3 was ‘neutral’). As per the four items representing emotional reactions, the mean response was 2.46 (*SD* = 1.05), which indicated that participants did not agree (i.e., answer option 2 was ‘disagree’). See Fig. 2 for an overview of all responses per item.

## 4 Discussion

In this randomized controlled trial, we explored potential reactivity effects of self-monitoring prolonged grief reactions five times per day for two weeks using experience sampling methodology (ESM) in recently bereaved people who expressed interest in grief treatment (i.e., a treatment-interested sample). The aim was to determine whether such self-monitoring would lead to (clinically relevant) changes in symptom severity levels of early prolonged grief disorder (PGD), post-traumatic stress disorder (PTSD), and depression on the group- as well as the individual-level.

The main finding was that, at the group-level, there was no reactivity effect, neither positive nor negative, of self-monitoring prolonged grief reactions multiple times a day in a treatment-interested sample of recently bereaved people. More specifically, we found no significant group differences between the ESM and waitlist-control condition in the severity of early PGD, PTSD, and depression symptoms when accounting for baseline symptom severity. Similarly, when examining changes in psychopathology levels at the individual-level from pre- to post-ESM in the form of clinically relevant change indices, we also found that the majority of participants did not report clinically relevant changes.

These findings contrast with the scarce and, notably, uncontrolled findings from previous ESM in the grief field, which suggested a potential positive reactivity effect (i.e., an alleviation of symptoms) from self-monitoring grief in daily life (Lenferink et al., 2022a; Mintz et al., 2023; for similar findings in daily diary studies see Aeschlimann et al., 2024; Buyukcan-Tetik et al., 2024; Eisma et al., 2024). However, the lack of a control group in these earlier studies suggests that their findings may be due to other factors, such as spontaneous improvement over time, consistent with natural remission after the loss of a loved one (cf. Reitsma et al., 2023). Our controlled findings suggest that assessing prolonged grief multiple times a day does overall not lead to changes in psychopathology levels, indicating that using ESM to assess prolonged grief in daily life does not result in a reactivity effect.

In its essence, this aligns with the intended use of ESM as a monitoring and assessment method rather than an intervention method (cf. Eisele et al., 2022). In contrast, ecological momentary interventions (EMIs) are specifically designed to induce changes and reactivity effects by incorporating personalized feedback and exercises during the ESM period and therefore go beyond passively monitoring (cf. Teixeira, 2022). Our findings align with previous studies outside the grief field, which report no reactivity effect in various behaviors or affective experiences from pre- to post-ESM (cf. Eisele et al., 2022; Ottenstein et al., 2024), but do report reactivity effects when using EMIs (e.g., Kramer et al., 2014). Therefore, our findings also suggest that ESM can serve as a useful research tool to study prolonged grief reactions in daily life without exacerbating symptoms, even in recently bereaved individuals. This conclusion is also supported by participants' feedback, who indicated that they found completing questions about their grief in the app to be a neutral experience, neither particularly beneficial nor emotionally troubling.

Nevertheless, although the overall group- and individual-level comparisons indicated no reactivity effects, a minority of our participants did experience a clinically significant improvement (i.e. 14 % for PGD severity, 4 % for PTSD, and 5 % for depression severity) following the two-week ESM self-monitoring, while even fewer participants experienced a deterioration in symptoms (i.e., 3 % for PGD severity, 3 % for PTSD, and 1 % for depression severity). Due to the very small number of participants who experienced a deterioration, we were only able to test predictors for improvement. Baseline PGD symptom severity was the only significant predictor. More specifically, the higher the baseline PGD symptom severity the higher the chance to experience a clinically relevant improvement in psychopathology levels compared to experiencing no change or a deterioration.

Thus, while self-monitoring prolonged grief reactions via ESM may not uniformly benefit all bereaved people, it potentially has a positive effect for those experiencing relatively high levels of early PGD symptoms three to six months following their loss. In other words, people who may already exhibit clinical levels of early prolonged grief, and thus are at higher risk of developing full-blown PGD at the 12-month post-loss cut-off (APA, 2022), could potentially benefit from an accessible ESM self-monitoring tool during the early months of grieving. However, these observations are based on secondary analyses with a small number of participants in the improvement group and therefore limited statistical power. Also, given that we do not have follow-up data at least 12 months post-loss, and the overall group findings of no reactivity effect after ESM, these interpretations should be considered provisional. A follow-up period assessing symptom severity at least 12 months after the loss could help in further understanding who is more likely to develop clinical levels of prolonged grief and to what extent (the reactivity effects of) self-monitoring prolonged grief reactions can help explain this development. Some may also argue that the observed improvement reflects natural recovery; however, consistent with recent trajectory studies (e.g., Djelantik et al., 2022; Lundorff et al., 2020), natural recovery of PGD within the first 6 months post-loss is highly unlikely, especially within two weeks. In sum, mental health professionals could consider incorporating ESM to help clients increase self-insight by tracking and reflecting on their grief experiences, potentially also using it as an ecological valid signaling tool to monitor deteriorations or improvements in the intensity of grief reactions. In addition, discussing with clients which factors may have caused changes in their momentary grief reactions, for instance based on contextual factors (such as where you were and what you were doing) assessed using ESM may provide further input to provide personalized therapeutic feedback (Pociūnaitė et al., 2024; von Klipstein et al., 2025). However, whether ESM, in contrast to EMIs, can, for some bereaved people actually lead to a clinically relevant improvement in psychopathology symptoms requires further study.

### 4.1 Strengths and limitations

Strengths of this study include the pre-registered randomized controlled design, the use of both group-level and individual-level analyses, and the detailed exploration of various factors potentially predicting clinically relevant changes in psychopathology severity. In addition, the assessment of participant's own perceptions of their experience with self-monitoring prolonged grief adds valuable insights into the acceptability of using ESM in the context of daily grief reactions. More generally, this study was the very first RCT in the grief field evaluating the effect of self-monitoring prolonged grief reactions in daily life using ESM.

At the same time, our findings should also be viewed in light of several limitations. Our sample consisted of a treatment-interested group of recently bereaved persons who directly indicated their willingness to participate in grief-related research. This self-selection bias can impact the generalizability of our findings. Similarly, our sample, although large for ESM studies (cf. Gabriel et al., 2019; Vachon et al., 2019), mainly consisted of women who lost a spouse or parent due to natural causes, and of which about half have received prior mental health support (in general or specifically related to their loss). Given previous research indicating that people bereaved due to unnatural, traumatic causes and who have lost a child have a considerably higher chance of developing persistent severe grief and other psychopathologies (Buur et al., 2024), our study should be replicated with these limitations in mind.

Another limitation could be the relatively short duration of the self-monitoring phase, i.e., two weeks. For a positive reactivity effect to occur, for example due to increased self-awareness and –insight (cf., Johnson et al., 2009), participants may need a more extensive time period of self-monitoring their grief reactions in daily life. Therefore, future studies could consider extending the ESM period to increase the possibility of observing clinically significant changes in symptom severity. Ideally, in line with previously discussed differences between ESM and EMI methods, future studies could also compare an (extended) ESM period with an active comparison group engaging in non-grief-related self-monitoring, as well as a separate EMI period with an active grief-related intervention (cf. Kramer et al., 2014; also see Goldberg et al., 2023). This would help further disentangle which aspects of intensive, repeated measures in daily life may be associated with clinical changes in (early) bereaved people, both with and without high initial prolonged grief reactions.

## 5 Conclusions

In summary, in this RCT, we found that, compared to a waitlist control condition, self-monitoring prolonged grief reactions five times per day for two weeks did not lead to changes in neither early PGD, PTSD, nor depression symptom severity. This means that overall, there seemed to be no reactivity effect, neither positive nor negative, to reporting on prolonged grief symptoms multiple times per day. However, individual-level analyses did suggest higher baseline PGD severity to be a predictor of clinically relevant improvement in bereavement-related outcomes, suggesting that self-monitoring might be particularly beneficial for those with higher initial levels of prolonged grief. Our findings, in line with previous results from uncontrolled ESM grief studies (Lenferink et al., 2022a; Mintz et al., 2023), suggest that ESM is a suitable and acceptable method for monitoring and assessing (early) prolonged grief experiences in daily life, both in scientific studies, as well as a potential self-management tool for bereaved people.

The following are the supplementary data related to this article.

Supplementary Fig. 1 Plotting the predicted probability of experiencing a clinically relevant improvement in bereavement-related outcomes (compared to no change or deterioration) by early Prolonged Grief Disorder (PGD) symptoms at baseline (T1). Supplementary Fig. 1

Supplementary tables Image 1

## Funding details

This publication is part of the project ‘Toward personalized bereavement care: Examining individual differences in response to grief treatment’ [ID: Vl.Veni.211G.065] of the research programme [NWO Talent Programme 2021 - Veni] which is financed by the Dutch Research Council (NWO) and awarded to Lonneke I.M. Lenferink.

## Declaration of competing interest

None.

## Footnotes

1 Results were comparable in terms of both significance and direction of effects to those originally reported in the manuscript. Specifically, the ANCOVAs did not show significant differences in early PGD, PTSD, or depression severity after the ESM period (*n* = 66) or waiting period (*n* = 89) when taking into account baseline severity (T1) as well as the number of completed ESM measurements (see Supplementary Table 2). These findings suggest that the level of missing within-person data did not substantially bias the reactivity estimates.
