# Supplementary material for: Exploring reactivity effects of self-monitoring prolonged grief reactions in daily life: A randomized waitlist-controlled trial using experience sampling methodology
Source: Internet Interv. 2025 Oct 2;42:100877. doi: 10.1016/j.invent.2025.100877 (PMC12524560; doi:10.1016/j.invent.2025.100877)
Supplement: Supplementary Fig. 1 — Plotting the predicted probability of experiencing a clinically relevant improvement in bereavement-related outcomes (compared to no change or deterioration) by early Prolonged Grief Disorder (PGD) symptoms at baseline (T1). [file mmc1.docx]

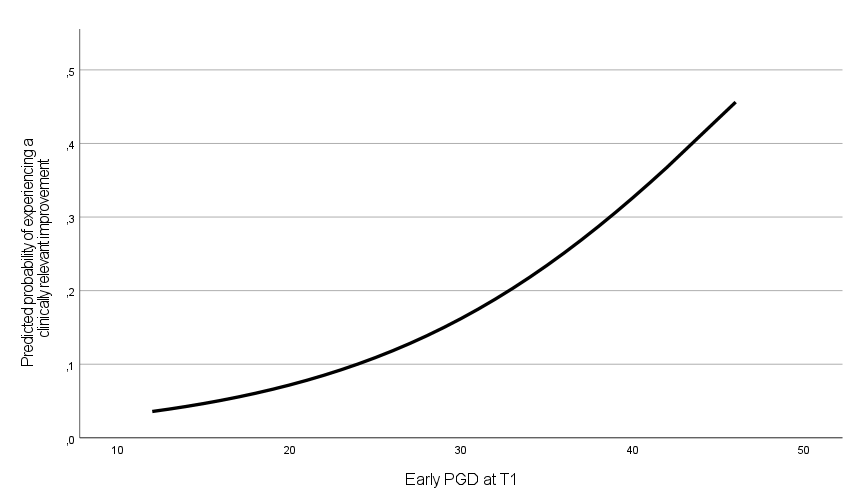


**Supplementary Figure 1. Plotting the predicted probability of experiencing a clinically relevant improvement in bereavement-related outcomes (compared to no change or deterioration) by early Prolonged Grief Disorder (PGD) symptoms at baseline (T1).**
