# Supplementary material for: Exploring reactivity effects of self-monitoring prolonged grief reactions in daily life: A randomized waitlist-controlled trial using experience sampling methodology
Source: Internet Interv. 2025 Oct 2;42:100877. doi: 10.1016/j.invent.2025.100877 (PMC12524560; doi:10.1016/j.invent.2025.100877)
Supplement: Supplementary file 2 — Supplementary tables [file mmc2.docx]

**Supplemental Materials**

| **Supplementary Table 1.** | | | | | | | | | | |
| --- | --- | --- | --- | --- | --- | --- | --- | --- | --- | --- |
| *Estimates for Background and Loss-related Characteristics and Psychopathology Symptom Levels Predicting Likelihood of Drop-out.* | | | | | | | | | | |
|  | *B* | | *SE* | | *p* | | *OR* | | 95% CI | |
| Covariate |  |  | |  | |  | | *LL* | | *UL* |
| Gender | .074 | .519 | | .886 | | 1.077 | | .389 | | 2.982 |
| Age | -.025 | .018 | | .163 | | .976 | | .942 | | 1.010 |
| Education | .194 | .412 | | .639 | | 1.214 | | .541 | | 2.722 |
| Time since loss | .055 | .039 | | .901 | | 1.005 | | .931 | | 1.085 |
| Kinship | .102 | .410 | | .804 | | 1.107 | | .495 | | 2.475 |
| Expectancy | -.092 | .130 | | .478 | | .912 | | .706 | | 1.177 |
| Cause of death | .222 | .530 | | .675 | | 1.249 | | .442 | | 3.530 |
| Current grief support | -.369 | .840 | | .660 | | .691 | | .133 | | 3.584 |
| Psychopathology-levels (T1) |  |  | |  | |  | |  | |  |
| PGD | -.024 | .028 | | .392 | | .976 | | .923 | | 1.032 |
| PTSD | -.009 | .017 | | .590 | | .991 | | .959 | | 1.024 |
| Depression | .045 | .041 | | .264 | | 1.046 | | .966 | | 1.133 |
| *Note*. The following variables were dichotomized: education ((applied) university vs. primary, secondary, and vocational education), kinship (spouse or child vs. other), cause of death (natural vs. unnatural). PGD = prolonged grief disorder; PTSD = posttraumatic stress disorder; T1 = pre-ESM or pre-waiting assessment; *B* = unstandardized beta; *SE* = standard error; OR = odds ratio; CI = confidence interval; LL = lower limit; UL = upper limit. | | | | | | | | | | |

| **Supplementary Table 2.** | | | | | | |
| --- | --- | --- | --- | --- | --- | --- |
| *Estimated Parameters for Analyses of Covariance Comparing the ESM and Waitlist Condition in the Completers Sample (n = 155) including the number of completed ESM measurements as covariate.* | | | | | | |
|  | **Early PGD at T2/T1b** | | **PTSD at T2/T1b** | | **Depression at T2/T1b** | |
|  | *B* | *SE* | *B* | *SE* | *B* | *SE* |
| Intercept | 6.26 | 2.12 | 5.55 | 2.28 | 0.38 | 1.07 |
| Condition | 0.52 | 0.78 | 0.39 | 1.20 | 0.76 | 0.53 |
| Symptom intensity at T1 | 0.76*** | 0.05 | 0.80*** | 0.05 | 0.71*** | 0.05 |
| Completed ESM measurements | -0.02 | 0.02 | -0.06 | 0.04 | 0.02 | 0.02 |
| *Note.* PGD = prolonged grief disorder; PTSD = posttraumatic stress disorder; T1 = pre-ESM or pre-waiting assessment; T1b = post-waiting assessment; T2 = post-ESM assessment; *B* = unstandardized beta; *SE* = standard error; **p* < .05, ***p* < .01, ****p* < .001. | | | | | | |

| **Supplementary Table 3.** | | | | | | |
| --- | --- | --- | --- | --- | --- | --- |
| *Estimated Parameters for Analyses of Covariance Comparing the ESM and Waitlist Condition in the Completers Sample (n = 155).* | | | | | | |
|  | **Early PGD at T2/T1b** | | **PTSD at T2/T1b** | | **Depression at T2/T1b** | |
|  | *B* | *SE* | *B* | *SE* | *B* | *SE* |
| Intercept | 3.53 | 1.98 | 2.12 | 2.13 | 0.28 | 0.95 |
| Condition | 0.72 | 0.74 | 0.16 | 1.14 | 0.58 | 0.51 |
| Symptom intensity at T1 | 0.76*** | 0.05 | 0.81*** | 0.05 | 0.70*** | 0.05 |
| *Note.* PGD = prolonged grief disorder; PTSD = posttraumatic stress disorder; T1 = pre-ESM or pre-waiting assessment; T1b = post-waiting assessment; T2 = post-ESM assessment; *B* = unstandardized beta; *SE* = standard error; **p* < .05, ***p* < .01, ****p* < .001. | | | | | | |

| **Supplementary Table 4.** | | | | | | | | | | | | | | | |
| --- | --- | --- | --- | --- | --- | --- | --- | --- | --- | --- | --- | --- | --- | --- | --- |
| *Estimates for Covariates Predicting Likelihood of Belonging to Group with Clinically Relevant Improvement* (*n* = 23) *Compared to No Change* (*n* = 104) *and of Belonging to Group with Clinically Relevant Deterioration* (*n* = 10) *Compared to No Change* (*n* = 104)*.* | | | | | | | | | | | | | | | |
| Improvement vs  No Change | | |  | | |  | | |  | | |  | |  | |
| Covariate | *B* | | | | | *SE* | | | *p* | | | *OR* | | 95% CI | |
|  |  | | | |  | | |  | | |  | | *LL* | | *UL* |
| Gender | 1.036 | | | | .778 | | | .183 | | | 2.817 | | .613 | | 12.943 |
| Age | .001 | | | | .019 | | | .985 | | | 1.000 | | .964 | | 1.038 |
| Education | .052 | | | | .472 | | | .912 | | | 1.054 | | .418 | | 2.656 |
| Time since loss | .023 | | | | .046 | | | .617 | | | 1.023 | | .935 | | 1.120 |
| Kinship | -.634 | | | | .494 | | | .200 | | | .531 | | .201 | | 1.398 |
| Expectancy | .217 | | | | .156 | | | .163 | | | 1.242 | | .916 | | 1.686 |
| Cause of death | -.354 | | | | .597 | | | .553 | | | .702 | | .218 | | 2.263 |
| Completed ESM moments | -.001 | | | | .028 | | | .963 | | | .999 | | .945 | | 1.055 |
| Current grief support | .788 | | | | .841 | | | .349 | | | 2.200 | | .423 | | 11.440 |
| Symptom-levels (T1) |  | | | |  | | |  | | |  | |  | |  |
| PGD | .090 | | | | .038 | | | **.019 *** | | | 1.094 | | 1.015 | | 1.180 |
| PTSD | .026 | | | | .020 | | | .190 | | | 1.026 | | .987 | | 1.067 |
| Depression | .095 | | | | .046 | | | **.039 *** | | | 1.100 | | 1.005 | | 1.204 |
| Self-insight total | -.020 | | | | .018 | | | .260 | | | .980 | | .946 | | 1.015 |
| Self-reflection subscale | -.020 | | | | .024 | | | .406 | | | .980 | | .934 | | 1.028 |
| Self-insight subscale | -.031 | | | | .033 | | | .341 | | | .969 | | .909 | | 1.034 |
| Deterioration vs  No Change | |  | |  | | |  | | |  | | |  | |  |
| Gender ^A^ | | - | | - | | | - | | | - | | | - | | - |
| Age | | -.018 | | .026 | | | .489 | | | .982 | | | .934 | | 1.033 |
| Education | | 1.808 | | 1.073 | | | .092 | | | 6.097 | | | .075 | | 49.926 |
| Time since loss | | .032 | | .066 | | | .633 | | | 1.32 | | | .907 | | 1.175 |
| Kinship | | 1.040 | | .718 | | | .147 | | | 2.830 | | | .693 | | 11.551 |
| Expectancy | | .124 | | .217 | | | .568 | | | 1.132 | | | .739 | | 1.734 |
| Cause of death ^A^ | | - | | - | | | - | | | - | | | - | | - |
| Completed ESM moments | | -.036 | | .038 | | | .346 | | | .965 | | | .896 | | 1.039 |
| Current grief support ^A^ | | - | | - | | | - | | | - | | | - | | - |
| Symptom-levels (T1) | |  | |  | | |  | | |  | | |  | |  |
| PGD | | -.018 | | .048 | | | .706 | | | .982 | | | .893 | | 1.080 |
| PTSD | | .016 | | .028 | | | .575 | | | 1.016 | | | .961 | | 1.074 |
| Depression | | .098 | | .065 | | | .131 | | | 1.103 | | | .971 | | 1.252 |
| Self-insight total | | -.011 | | .026 | | | .661 | | | .989 | | | .939 | | 1.041 |
| Self-reflection subscale | | .017 | | .040 | | | .664 | | | 1.017 | | | .941 | | 1.099 |
| Self-insight subscale | | -.060 | | .045 | | | .183 | | | .941 | | | .861 | | 1.029 |
| *Note*. The following variables were dichotomized: education ((applied) university vs. primary, secondary, and vocational education), kinship (spouse or child vs. other), cause of death (natural vs. unnatural). PGD = prolonged grief disorder; PTSD = posttraumatic stress disorder; T1 = pre-ESM or pre-waiting assessment; *B* = unstandardized beta; *SE* = standard error; OR = odds ratio; CI = confidence interval; LL = lower limit; UL = upper limit.  ^A^ = analysis not possible because no cases present (no men/no unnatural death/no current grief support in the deterioration group) | | | | | | | | | | | | | | | |
